# Knockout of *MULTI-DRUG RESISTANT PROTEIN 5* Genes Lead to Low Phytic Acid Contents in Oilseed Rape

**DOI:** 10.3389/fpls.2020.00603

**Published:** 2020-05-26

**Authors:** Niharika Sashidhar, Hans J. Harloff, Christian Jung

**Affiliations:** Plant Breeding Institute, Agrar und Ernährungswissenschaftliche Fakultät, Christian-Albrechts-University of Kiel, Kiel, Germany

**Keywords:** ATP binding cassette, *Brassica napus*, *BnMRP5*, lpa, phosphorous, TILLING, EMS, mutant

## Abstract

Understanding phosphate uptake and storage is interesting to optimize the plant performance to phosphorus fluctuations. Phytic acid (PA) is the major source of inorganic phosphorus (Pi) in plants. Genetic analyses of PA pathway transporter genes (*BnMRP5*) and their functional characterization might provide clues in better utilizing the available phosphate resources. Furthermore, the failure to assimilate PA by monogastric animals results in its excess accumulation in manure, which ultimately causes groundwater eutrophication. As a first step toward breeding low PA mutants in oilseed rape (*Brassica napus* L.), we identified knockout mutants in PA biosynthesis and transporter genes. The obtained M_3_ single mutants of *Bn.MRP5.A10* and *Bn.MRP5.C09* were combined by crossing to produce double mutants. Simultaneously, crosses were performed with the non-mutagenized EMS donor genotype to reduce the background mutation load. Double mutants identified from the F_2_ progeny of direct M_3_ crosses and BC_1_ plants showed 15% reduction in PA contents with no significant differences in Pi. We are discussing the function of *BnMRP5* paralogs and the benefits for breeding *Bnmrp5* mutants in respect to low PA, yield, and stress tolerances.

## Introduction

Phosphorus is an essential macronutrient required for plant development. In plants, numerous transporters such as PHT, SPX-MFS, and phosphate antiporters facilitate the uptake of phosphorus from the soil and its mobilization across various tissues (Kopriva and Chu, 2018). The up taken phosphorus is utilized in various metabolic processes and is finally stored in seed vacuoles in the form of phytic acid (PA; also referred as inositol hexakis phosphate), which is readily available for seedling development. The PA stored in vacuoles is referred to as phytin, which is a complex salt of divalent ions. In dry mature seeds of various plant species, up to 80% of total phosphorus is stored as PA (Raboy et al., 2000). Therefore, low PA mutants are desirable for a reduced application of external phosphorus in the form of fertilizers. Furthermore, they could help to decrease the dependency on non-renewable rock phosphates (Lott et al., 2007). In this regard, PA biosynthetic genes have been knocked out in different plant species to obtain low PA mutants with simultaneous increase of inorganic phosphorus (Pi) (Sparvoli and Cominelli, 2015). In some cases, the knockout of genes involved in PA biosynthesis has proven to have adverse effects on plant performances such as poor seed set, delayed germination, and low yield (Shi et al., 2007). Therefore, targeting the transporters that are accumulating PA in seeds gained more attention.

A multi-drug resistant protein (MRP5), acting as a tonoplast transporter was shown to have high affinity to PA (Nagy et al., 2009). MRP5 is a member of the ATP binding cassette (ATP) super family and belongs to the ABCC subfamily (Kang et al., 2011). The protein has two transmembrane domains and two nucleotide-binding domains encasing the walker A, B, and C motifs (Verrier et al., 2008). It has been shown that in Arabidopsis the transport of PA is strictly dependent on ATP (Nagy et al., 2009) whereas in rice knockout of *SULTR-like phosphorus distribution transporter* (SPDT) genes lead to 25–32% reduced PA contents in seeds (Yamaji et al., 2016). This indicates that the vacuolar loading of phosphorus in seeds is not limited to the MRP5 protein but can also be achieved by other transporters. Furthermore, the accumulation of PA in the vacuoles depends on the availability of Pi, as it is highly important to maintain the ratio of vacuolar to cytoplasmic Pi for overall phosphorus homeostasis (Pratt et al., 2009).

Oilseed rape (*Brassica napus* L.) is the third most important oil crop in the world and its seed meal is rich in proteins and amino acids (Gacek et al., 2018). However, anti-nutritive compounds such as glucosinolates and tannins together with a high fiber content impede its use as a valuable protein source for human and animal feed. Also, PA has an anti-nutritive effect because it is a strong chelator of positive ions like magnesium, potassium, zinc, and iron, thereby reducing their bioavailability in monogastric animals. Moreover, it contributes to phosphate pollution because PA cannot be digested due to lack of phytases thereby leading to high P concentrations in the manure. Several knockout and knockdown mutants of PA transporter genes were identified in cereal crops such as maize, rice, wheat, and barley. Their seed PA contents were reduced by 32–90% with a simultaneous increase of Pi (Dorsch et al., 2003; Shi et al., 2007; Xu et al., 2009; Panzeri et al., 2011; Bhati et al., 2016). In addition, *MRP* mutants were identified from common bean and soybean, which also showed a substantial reduction of PA contents by up to 90% (Wilcox et al., 2000; Shi et al., 2007; Campion et al., 2009).

We expected that *MRP* genes display a similar function in rapeseed. Therefore, we aimed to select *MRP* mutants with reduced PA seed storage. We identified *MRP* orthologs in the rapeseed genome and used the sequence information to select EMS mutants by a novel TILLING by Sequencing (TbyS) approach (Sashidhar et al., 2019). Only double mutants showed a significant reduction in seed PA content. These mutants will be important for breeding low PA rapeseed varieties.

## Materials and Methods

### Plant Material and Growth Conditions

The rapeseed winter type variety Express 617 was used to study PA and Pi accumulation in developing seeds. Plants were grown in a greenhouse in 11 cm × 11 cm pots at 22°C under long day conditions (16 h of light and 8 h of dark). No external phosphorus was added to the soil. Three weeks old plants were vernalized for 8 weeks at 4°C (16 h light and 8 h dark) and after 8 weeks plants were transferred back to 22°C (16 h light and 8 h dark) until harvest. At each time point, five plants were harvested. During flowering, young buds were emasculated and hand-pollinated with Express 617 pollen to mark the day of pollination. For PA and Pi extractions, developing seeds were collected 15, 25, 35, 45, and 55 days after pollination (DAP) and frozen in liquid nitrogen. Samples were kept at −80°C until extraction.

**FIGURE 1:**
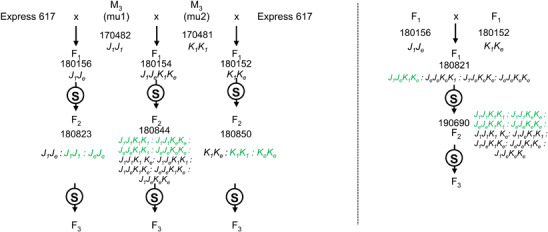
Crossing scheme for the production of *BnMRP5* single and double mutants. The seed codes are written above each genotype (see Table 3 for respective allele codes). The genotypes highlighted in green were selfed to produce F_3_ seeds, which were used for phenotyping; “S”: selfing; *Bn.MRP5.A10* and *Bn.MRP5.C05* mutations are indicated as mu1 and mu2, respectively.

M_3_ seeds were obtained from the breeding company Norddeutsche Pflanzenzucht Hans-Georg Lembke KG (Hohenlieth, Germany). M_3_ plants homozygous for the *Bn.MRP5.A10* allele (M_3_ seed code 170482) were crossed with homozygous *Bn.MRP5.C05* mutants (M_3_ seed code 170481) to produce F_1_ plants. Simultaneously, homozygous M_3_ plants from each family were crossed with non-mutagenized Express 617 plants to obtain F_1_ offspring with reduced background mutation load. All F_1_ plants were selfed to produce F_2_ seeds (Figure 1).

### Identification of MRP5 Genes and Mutant Screening

In Arabidopsis, the MRP5 protein is encoded by one gene (At1g0420), which was used for retrieving all paralogous genes from *B. napus* using the Genoscope browser^1^. Genes having high sequence identity to the Arabidopsis genes were considered as true paralogous genes. Protein domains were identified using the pFam database search^2^. All hits were confirmed using TAIR^3^, NCBI annotations^4^ and BRAD databases^5^.

We have used TbyS for screening the mutations as described in Sashidhar et al. (2019). Two *BnMRP5* paralogs were chosen for identifying the putative loss of function mutations. Four primer pairs were used to screen amplicons 1414–1509 bp in size. Sanger sequencing was used to confirm the mutations in the M_2_ generation with the help of paralog specific primers.

Mutation frequencies (*F*) were calculated based on the mutations per M_1_ plant (Harloff et al., 2012):

$$F\,\left[1/\mathrm{kb}\right]=1/\left(\frac{\left(\mathrm{amplicon}\,\mathrm{size}\,\left[\mathrm{bp}\right]\right)*\left(\mathrm{number}\,\mathrm{of}\,M_{1}\,\mathrm{plants}\right)}{\left(\mathrm{number}\,\mathrm{of}\,\mathrm{mutations}\right)*1,000}\right)$$

### Nucleic Acid Isolation, PCR, and RT-qPCR

For genotyping experiments, DNA was isolated from the leaves using the CTAB protocol (Rogers and Bendich, 1985) with minor modifications. PCR was performed using DNA aliquots of 2 μl as a template for amplifying the genes (92°C: 3 min, [92°C: 30 s, 58–63°C: 30 s, 72°C: 65 s] 36x, 72°C: 5 min). Amplicon lengths were verified on 1% agarose gels and the presence of mutations was confirmed by Sanger sequencing (Table 1).

**TABLE 1:** Primers used for genotyping and expression analysis.

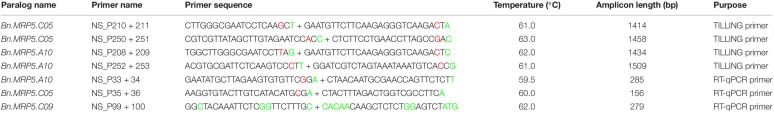

*Green letters indicate SNPs between paralogs and red letters indicate modified SNP to increase the specificity. Primer design is done according to Liu et al. (2012).*

For expression analysis, the winter type line Express 617 was grown in the greenhouse as described above. Five plants were used for expression analysis. Immature flower buds were emasculated followed by hand pollination to ensure the day of pollination. Seed samples of 50 mg were harvested at 15, 25, 35, and 45 DAP and were shock frozen in liquid nitrogen. RNA was isolated using the peqGOLD RNA isolation kit (PEQLAB Biotechnologie GmbH, Erlangen, Germany) according to the manufacturer’s protocol. DNase digestion was performed after RNA isolation using the peqGOLD DNase I Digest Kit (PEQLAB Biotechnologie GmbH, Erlangen, Germany) to remove genomic DNA. cDNA first strand synthesis was carried out using a first strand cDNA synthesis kit (ThermoFisher Scientific Inc., Waltham, MA, United States) according to the manufacturer’s protocol. RT-qPCR was performed using Platinum^®^ SYBR^®^ Green qPCR SuperMix-UDG with ROX (Invitrogen, Karlsruhe, Germany) on a CFX96 Real-Time PCR detection system (Bio-Rad, Hercules, CA, United States) in a volume of 20 μl. *BnActin2* was used as a reference gene for normalization. Relative expression was calculated using the ΔC_*t*_ method (Table 1).

### Inorganic Phosphorus and Phytic Acid Measurements

Dry seeds of 200 mg were ground into fine powder and PA was extracted in three replicates of 50 mg according to Matthäus et al. (1995) with minor modifications. PA was measured by HPLC according to Rounds and Nielsen (1993) calibrating with a PA standard (Sigma P-8810). For determining Pi contents, 50 μl of the purified column extracts was mixed with 500 μl of coloring reagent [10% w/v of ascorbic acid (Roth-Art.Nr.3666.1) and 5% w/v of ammonium molybdate (Roth, Art. No.3525.2)] adjusted to 1.5 ml with double distilled water and incubated at 40°C for 1 h. The samples were measured against a reagent blank buffer in a spectrophotometer at 655 nm. A calibration curve ranging from 0 to 100 nmol was used for both PA and Pi measurements with standards for Pi and PA, respectively.

### Statistical Analysis

Data obtained from PA and Pi measurements were used for statistical analysis. Significance was calculated using ANOVA in R 3.6.0 and the MulticomView package and *post hoc* test was performed by using Tukey’s multiple comparison test; *p* = 0.05. Standard deviation was calculated between five biological samples along with their three technical repeats.

## Results

### Dynamics of Phytic Acid and Inorganic Phosphorous Accumulation in Winter Type Rapeseed

**FIGURE 2:**
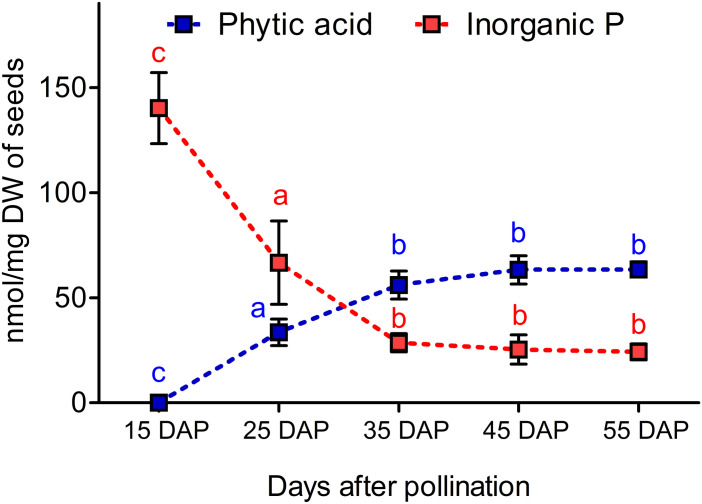
Dynamics of phytic acid (PA) and inorganic phosphorus (Pi) accumulation during seed maturation in the winter type Express 617. Plants were grown in a greenhouse under 16 h of light and 8 h of dark at 22°C. Five plants were used as biological replicates for PA and Pi measurements. Error bars denote the SD among five biological samples along with three technical repeats. Statistical significance was calculated using ANOVA and grouping was done using a Tuckey test. Different letters indicate statistical significance.

Seed samples from 15, 25, 35, 45, and 55 DAP were used to determine the dynamic changes of PA and Pi in developing seeds. We observed that PA was not measurable at 15 DAP, but it accumulated significantly from 15 to 35 DAP. In contrast, Pi contents decreased significantly from 15 to 35 DAP. The coordinated decrease of Pi and increase of PA might indicate that most of the Pi is assimilated to PA between 35 and 45 DAP (Figure 2).

### Searching the Rapeseed Genome for *MRP5* Orthologs

We used the Arabidopsis *AtMRP5* sequence as query to search the Brassica Genoscope database. Four rapeseed sequences were identified as putative paralogous genes of *AtMRP5* (Table 2). All genes showed Walker A, B, and C motifs, which were encased by the transmembrane domain (Supplementary Figure S1). The BLAT results from Genoscope browser showed that *Bn.MRP5.A09* had a lower homology and also a shorter protein sequence when compared to the three other proteins. Upon protein alignment with AtMRP5, we found that Bn.MRP5.A09 lacked most of the transmembrane domain regions, indicating that this protein might lack a membrane transport function for PA. Therefore, we excluded *Bn.MRP5.A09* from our analyses (Supplementary Figure S2).

**TABLE 2:** Gene structure and amino acid similarity of four *MRP5* paralogs in *Brassica napus* L.

**Arabidopsis gene**	***B. napus* sequence annotation**	***B. napus* paralogs**	**Transcript length (bp)**	**Exon/intron structure**		**Protein size (aa)**	Amino acid sequence identity with *AtMRP5* (%)*
				**Exons**	**Introns**		
At1g0420	*BnaA10g02400D*	*Bn.MRP5.A10*	5284	9	8	1509	93.0
	*BnaC05g02300D*	*Bn.MRP5.C05*	5278	9	8	1510	93.6
	*BnaC09g10390D*	*Bn.MRP5.C09*	5399	12	11	1436	85.3
	*BnaA09g10230D*	*Bn.MRP5.A09*	4168	13	12	1071	84.7

Transcript length is from start to stop codon. *% identity is obtained by using BLAT function from the Genoscope browser.

**FIGURE 3:**
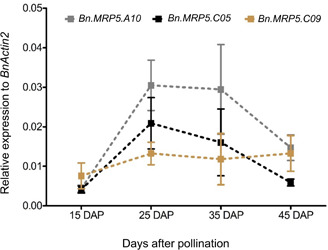
Expression profiles of three *BnMRP5* genes in Express 617 at seed filling stages. Plants were grown at 16 h of light and 8 h of dark at 22°C. Seed samples from four developmental stages of Express 617 were harvested at 15, 25, 35, and 45 DAP. The expression values were normalized to the housekeeping gene *BnActin2*. Error bars indicate SD of five biological samples along with three technical repeats.

Messenger RNA abundance was measured in developing seeds of Express 617 and C_*t*_ values were normalized to the housekeeping gene *BnActin2*. The transcriptional activities of all three genes strongly increased between 15 and 25 DAP. They peaked between 25 and 35 DAP and decreased 45 DAP (Figure 3). Finally, the two highest expressed paralogs (*Bn.MRP5.A10*, *Bn.MRP5.C05*) were used for mutation screening in the EMS mutant population.

### Identification of EMS Mutants and Production of Double Mutants

**FIGURE 4:**
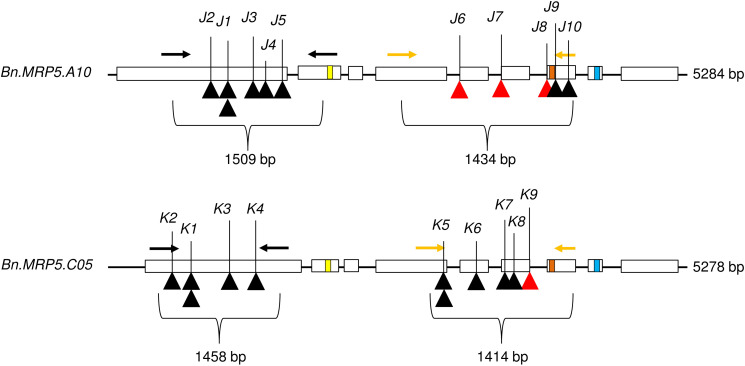
Exon–intron structure showing the positions of the mutations. Exons are shown as white boxes. Primer pairs used for amplicon sequencing are shown as black and yellow arrows. C motif, Walker A, and Walker B motif are shown as yellow, orange, and blue boxes, respectively. The C motif is also known as ABC signature motif. Stop codon and splice site mutations are shown as black and red triangles, respectively. The mutant alleles are indicated by respective numbers (see Table 3).

We applied a new TbyS protocol to search for EMS mutants in the Express 617 mutant population (Sashidhar et al., 2019). We screened for mutations within the translated regions of *Bn.MRP5.A10* (5284 bp) and *Bn.MRP5.C05* (5278 bp). The amplicons used for TbyS covered up to 60% of the coding regions of *Bn.MRP5.A10* and *Bn.MRP5.C05*, including the Walker A and C motifs of the genes (Figure 4). As a result, eight stop codon and three splice site mutations were found in *Bn.MRP5.A10* while 10 stop codon and one splice site mutation were found in *Bn.MRP5.C05* (Table 3). All mutations were confirmed by Sanger sequencing confirming the reliability of the TbyS strategy (Sashidhar et al., 2019). For ease of understanding, mutations in *Bn.MRP5.A10* and *Bn.MRP5.C05* were assigned with the allele code *J* and *K*, respectively. For phenotyping, we chose the alleles *J*_1_ and *K*_1_, as these mutations were at the beginning of the gene and the translation should result in a loss of all the important protein domains (Table 3 and Figure 4).

**TABLE 3:** All *Brassica napus BnMRP5* mutant alleles detected by TILLING by sequencing (TbyS).

**Gene**	**Mutation type**	**Mutation site (bp)^§^**	**Allele^a^**	**Mutation**	**Effect on protein sequence**
*Bn.MRP5.A10*	Non-sense	1077^$^	*J*_1_	G to A	W379*
*Bn.MRP5.A10*	Non-sense	1072	*J*_2_	C to T	Q378*
*Bn.MRP5.A10*	Non-sense	1242	*J*_3_	G to A	W414*
*Bn.MRP5.A10*	Non-sense	1260	*J*_4_	G to A	W420*
*Bn.MRP5.A10*	Non-sense	1561	*J*_5_	C to T	Q521*
*Bn.MRP5.A10*	Splice site	3557	*J*_6_	G to A	–
*Bn.MRP5.A10*	Splice site	4093	*J*_7_	G to A	–
*Bn.MRP5.A10*	Non-sense	4152	*J*_8_	G to A	W1216*
*Bn.MRP5.A10*	Non-sense	4271	*J*_9_	G to A	W1255*
*Bn.MRP5.A10*	Splice site	4390	*J*_10_	G to A	–
*Bn.MRP5.C05*	Non-sense	432	*K*_2_	G to A	W144*
*Bn.MRP5.C05*	Non-sense	628^$^	*K*_1_	C to T	Q210*
*Bn.MRP5.C05*	Non-sense	1174	*K*_3_	C to T	Q392*
*Bn.MRP5.C05*	Non-sense	1244	*K*_4_	G to A	W415*
*Bn.MRP5.C05*	Non-sense	3487^$^	*K*_5_	C to T	R1044*
*Bn.MRP5.C05*	Non-sense	3762	*K*_6_	C to T	Q1116*
*Bn.MRP5.C05*	Non-sense	4206	*K*_7_	C to T	Q1239*
*Bn.MRP5.C05*	Non-sense	4245	*K*_8_	C to T	Q1252*
*Bn.MRP5.C05*	Splice site	4299	*K*_9_	G to A	–
*Bn.MRP5.A10*	–	–	*J*_*e*_	–	–
*Bn.MRP5.C05*	–	–	*K*_*e*_	–	–

^§^Mutation site from the start codon. ^*a*^A single letter code was used to display M_2_ genotypes where “e” denotes the allele of non-mutagenized Express 617 donor variety. ^$^Two plants from the EMS population had similar mutation. *Indicates a stop codon.

It was known from previous studies that single mutants in rapeseed often do not display a new phenotype due to gene redundancy. Moreover, EMS mutants suffer from a huge number of background mutations, which severely hampers the phenotypic evaluation (Braatz et al., 2018; Shah et al., 2018). Therefore, we initiated a crossing program to produce double mutants and backcross generations with reduced mutation load. Homozygous M_3_ mutants were crossed with non-mutated Express 617 to obtain F_1_ seeds. Then, the F_1_ plants carrying mutations in both the selected copies were crossed with each other (Figure 1) and F_2_ populations were generated to be used for PA measurements. In the F_2_ generation, segregation ratios were as expected for mono (3:1) and digenic (15:1) segregation (Table 4).

**TABLE 4:** Genotypic segregation for *Bn.MRP5.A10* and *Bn*.*MRP5.C05* in 4 F_2_ populations.

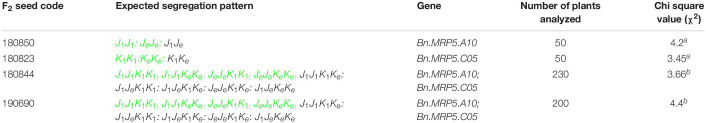

*^*a*^3:1 segregation, χ^2^ < 5.99, at *p* = 0.05, df = 2. ^*b*^15:1 segregation, χ^2^ < 7.82, at *p* = 0.05, df = 3. Genotypes shown in green were used for phenotyping experiments.*

### *Bnmrp5* Double Mutants With Reduced Phytic Acid Seed Content

**FIGURE 5:**
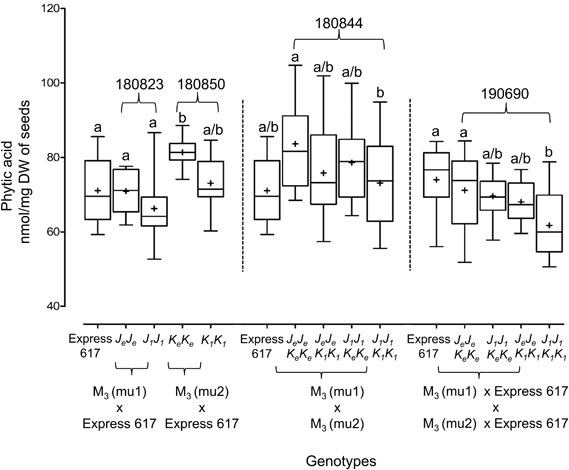
Phenotypic analysis of *Bnmrp5* mutants. Box plot of phytic acid contents in seeds of *Bnmrp5* mutants. Statistical analysis was performed using ANOVA test at *p* < 0.05 and grouping was done using the Tukey test. Same letters indicate no significant differences. The non-mutated Express 617 alleles are indicated by “e.” *Bn.MRP5.A10* and *Bn.MRP5.C05* mutations are indicated as mu1 and mu2, respectively. Seed codes of F_2_ families are given above the brackets. Means are indicated by “+.” Allele codes are given in Table 3.

**FIGURE 6:**
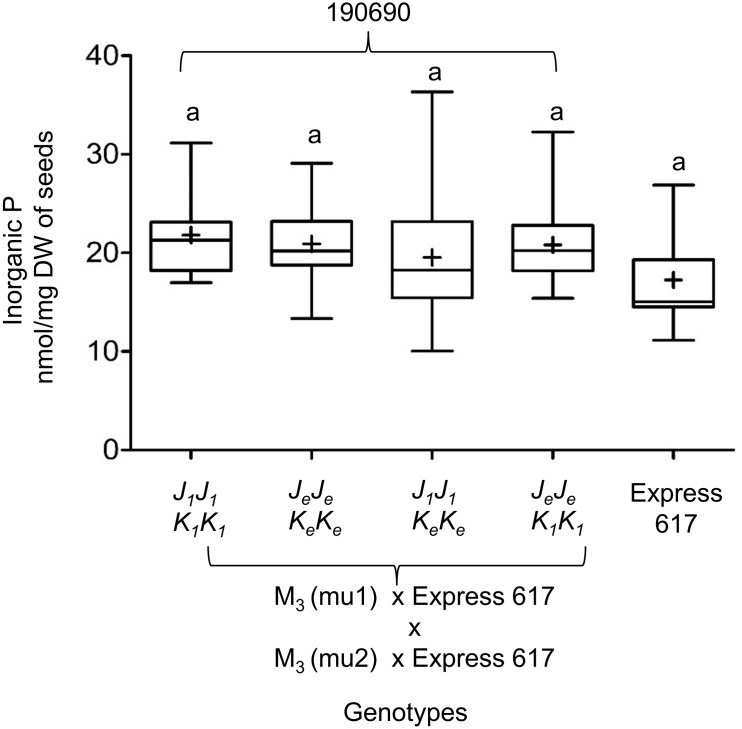
Inorganic P (Pi) measurements in *Bnmrp5* double mutants of the F_2_ family 190690. The boxplot represents Pi measurements in five plants for each genotype from matured seeds. Of each plant, three technical replicates were taken. Means are indicated by “+.” Statistical analysis was performed using ANOVA test at *p* < 0.05 and grouping was done using the Tukey test. Same letters indicate no significant differences between the samples. The non-mutated Express 617 alleles are indicated by “e.” *Bn.MRP5.A10* and *Bn.MRP5.C05* mutations are indicated as mu1 and mu2, respectively Allele codes are given in Table 3.

We measured the effect of single and double mutations with full and reduced mutation load. Plants from M_3_, F_2_, and BC_1_ generations were grown in the greenhouse. Express 617 and mutant offspring of Express 617 (suffix “*e*”) were used as controls. Single mutants from the two F_2_ families, 180823 (*J_1_J_1_*) and 180850 (*K_1_K_1_*), showed no significant reduction in PA contents when compared to both Express 617 controls (Figure 5). However, double mutants obtained from F_2_ family 180844 (*J_1_J_1_K_1_K_1_*) showed significant reduction in comparison to the mutant control plants carrying homozygous Express 617 alleles (*J_*e*_J_*e*_K_*e*_K_*e*_*) but not to non-mutagenized Express 617 (Figure 6). This could be explained due to a high mutation load. Therefore, we expected clear phenotypic effects in the double mutants backcrossed with Express 617 (see Figure 1). As a result, we observed a significant PA reduction in backcrossed double mutants from the F_2_ population 190690 in comparison to the internal control plants (*J_*e*_J_*e*_K_*e*_K_*e*_*) as well as to the Express 617 plants. We concluded that a reduction of 15% was due to the underlying mutant alleles and not due to the background mutation load. Indeed, a similar reduction was observed in the double mutants of the 180844 family when compared to internal control plants (*J_*e*_J_*e*_K_*e*_K_*e*_*), indicating that the observed reduction of 15% PA is due to the two mutated alleles. In addition, we measured the Pi contents in matured seeds of the 190690 progeny to see if the reduced PA content had any effect on Pi accumulation. We did not find a significant increase in double mutants as compared to control plants (Figure 6). Besides, plant height, silique number per plant, and thousand-kernel weight were not significantly different in the mutants, indicating that the low PA was not affecting the overall plant performance (Table 5).

**TABLE 5:** Evaluation of pleiotropic effects in F_3_ seeds of *Bnmrp5* mutants.

**F_2_ seed code**	**Generation**	**Genotype**	**TKW (g)**	**Plant height (cm)**	**Silique number/plant**
180844	M_3_(mu1) × M_3_(mu2)	*J_1_J_1_K_1_K_1_*	3.290.33	93.069.57	3911.98
		*J_1_J_1_K_*e*_K_*e*_*	3.370.10	101.809.12	427.08
		*J_*e*_J_*e*_K_1_K_1_*	3.320.38	105.009.33	3710.30
		*J_*e*_J_*e*_K_*e*_K_*e*_*	3.280.14	103.203.56	399.80
		Express 617	3.300.22	118.026.12	426.08
180823	M_3_(mu1) × Express 617	*J1J1*	2.610.16	91.538.56	465.08
		*JeJe*	2.370.20	98.884.60	527.10
180850	M_3_(mu2) × Express 617	*K_1_K_1_*	3.240.32	97.508.35	399.08
		*K_*e*_K_*e*_*	3.090.60	97.458.40	466.50
190690	[M_3_(mu1) × Express 617) × (M_3_(mu2) × Express 617]	*J_1_J_1_K_1_K_1_*	2.500.15	84.803.65	404.60
		*J_1_J_1_K_*e*_K_*e*_*	2.150.14	86.202.13	4512.20
		*J_*e*_J_*e*_K_1_K_1_*	2.670.40	90.208.25	408.79
		*JeJ_*e*_K_*e*_K_*e*_*	2.380.15	83.663.44	478.28
		Express 617	2.590.49	89.455.40	495.23

*Plants were grown under 16 h light and 8 h dark in 11 × 11 cm pots at 22°C for all measurements. Five plants for each genotype were used for measuring TKW, plant height, and silique number. *Bn.MRP5.A10* and *Bn.MRP5.C05* mutations are indicated as mu1 and mu2, respectively.*

## Discussion

In this study, we describe low PA mutants in oilseed rape by targeting *BnMRP5* PA transporter genes. Three paralogs were expressed during seed development, two showed a maximum expression between 25 and 35 DAP similar to the expression of MRP5 genes in siliques of Arabidopsis (*AtMRP5*, Kim and Tai, 2011) and common bean (*PvMRP1*, measured 6 and 24 days after flowering (Panzeri et al., 2011). The expression pattern of the paralogs was in line with the PA and Pi accumulation pattern from Express 617. Shortly after 35 DAP, PA storage reached its maximum and Pi depletion its minimum suggesting a major incorporation of Pi into PA until 35 DAP.

The double mutants obtained displayed up to 15% reduced PA contents. One of the major concerns regarding EMS mutants is their high mutation load. This problem became obvious when comparing primary (non-backcrossed) mutants and double mutants with their non-mutagenized parent Express 617. Therefore, we could not observe significant differences between primary double mutants (*J_1_J_1_K_1_K_1_*, F_2_ population 180844) and Express 617. However, double mutants once backcrossed with Express 617 (F_2_ population 190690) had significantly reduced PA contents compared to their mutant siblings (*J_*e*_J_*e*_K_*e*_K_*e*_*) and compared to Express 617.

We expected that the two knockout mutants resulted in a truncated protein, where the Walker A, B, and C motifs were completely lost, thereby leading to a failure to transport PA. However, the reduction in PA content was somewhat lower as in other studies with maize, rice, wheat, barley, Arabidopsis, soybean, and common bean where reductions between 32 and 90% have been reported (Sparvoli and Cominelli, 2015). One reason for a relatively lower reduction in our study could be that the third gene might partially contribute to the compensation of the phenotypic effect in the double mutant. In rice it has been shown that a mutation in *OsPLD*α*1* caused a reduction of PA in seeds, however, in the mutant other *PLD* genes were upregulated, indicating that the other PLD proteins can in part compensate the mutation in *OsPLD*α*1* (Khan et al., 2019). This genetic compensation response has first been described after mutant mRNA degradation for genes with sequence similarities to the mutated genes in zebrafish (El-Brolosy et al., 2019). Whether this applies also for the mutation of the two *MRP5* genes we have studied will be an interesting direction for further studies.

In polyploids, the simultaneous knockout of multiple genes is required to obtain a phenotypic effect (Wang et al., 2014; Del Pozo and Ramirez-Parra, 2015). In wheat, a reduction in PA content by “only” 22% was observed when all three paralogous genes of *TaABCC5* were downregulated by RNAi (Bhati et al., 2016). Therefore, this study and our study might indicate the existence of alternative transporters in polyploids, which might compensate for the loss of knockout mutations in MRP5 proteins. A study in common bean has shown that loss of *PvMRP1* was compensated by *PvMRP2*. However, compensation was not observed in seeds but in leaf tissues (Cominelli et al., 2018). In addition, reduced PA seed contents in knockout mutants of *OsSPDT* in rice, an SPDT (Yamaji et al., 2016), suggest its contribution to PA loading in seeds (Sacchi and Nocito, 2019). Since, loss of function mutants in PA transporters were never identified before in oilseed rape, the hypothesis that other MRP transporters compensate the knockout effects need to be further validated for instance by additional mutations in the third paralog *Bn.MRP5.C09*. Moreover, they can be crossed with *bn2-pgk2* mutants which we have been recently identified (Sashidhar et al., 2019). Like the *bnmrp5* mutants, *bn2-pgk2* mutants did not show any trade off effects regarding agronomically important characters. Therefore, creating multiple mutants in both gene families could be a promising option to further reduce PA seed contents in oilseed rape.

Knock out mutants of *MRP5* in Arabidopsis showed a major reduction in PA contents. Therefore, we assumed that this might also be the case in *BnMRP5* mutants of the close relative *B. napus*. PA transporter mutants in various species do have higher inositol kinases (Vip/VIH) referred to as inositol hexakis phosphate kinases (Desai et al., 2014), which synthesize InsP7 (also referred as PP-InsP_5_) and InsP8 (also referred as 1,5 PP_2_-InsP_4_; Supplementary Figure S3). For instance, the knockout mutants of *atmrp5* and *zmmrp4* showed an increased accumulation of InsP7. Loss of function in MRP5 might therefore lead to an increased concentration of InsP6 (or PA) in the cytosol, which can be used as a substrate by the inositol hexakis phosphate kinases to produce InsP7 or InsP8, resulting in a lower PA storage rate. Accumulating InsP7 and InsP8 might be beneficial to plants as they possess energy-rich pyrophosphate moieties having a comparable free energy of hydrolysis to ATP (Hand and Honek, 2007; Desai et al., 2014). It was shown in Arabidopsis that InsP7/InsP8 binds to SPX domains, which are crucial in phosphate sensing and in regulating Pi homeostasis (Jung et al., 2018; Ried et al., 2019). Thereby with an increased accumulation of such molecules, the yield capacity can be improved and also fertilizer demands might be reduced (Williams et al., 2015). Finally, inositol pyrophosphates are regulating the jasmonic acid-based defense mechanisms, which might be beneficial for an improved growth under stress conditions (Laha et al., 2015).

*AtMRP5* Arabidopsis mutants have an elevated drought tolerance due to a reduced ABA sensitivity resulting in stomatal closure (Gaedeke et al., 2001; Klein et al., 2003). In *atmrp5* mutants, failure to transport PA into vacuoles resulted in reduced K^+^ uptake into the guard cells by inhibiting K^+^ inward rectifying channels (Nagy et al., 2009). The stomatal closure might reduce water loss from leaves and might foster plants to cope with drought stress and it was shown that *atmpr5* mutants showed reduced water uptake and survived longer than the wild type plants under low water levels (Klein et al., 2003). However, as we observed only 15% reduction of PA, a complete knockout of *BnMRP5* genes in future might pave the way for a comprehensive analysis of *BnMRP5* genes under drought stress. In conclusion, the mutants presented here are important for breeding low PA rapeseed varieties. Moreover, they can be utilized in exploring phosphorous sensing and homeostasis mechanisms.

## Data Availability Statement

All datasets generated for this study are included in the article/Supplementary Material.

## Author Contributions

NS planned, performed, and analyzed the experiments and wrote the manuscript. HH and CJ designed the study, supervised the experiments, and revised the manuscript. All authors read and approved the final manuscript.

## Conflict of Interest

The authors declare that the research was conducted in the absence of any commercial or financial relationships that could be construed as a potential conflict of interest.

## Footnotes

1 http://www.genoscope.cns.fr/brassicanapus/

2 http://pfam.xfam.org/

3 https://www.arabidopsis.org/servlets/Search?type=gene&action=new_search

4 https://www.ncbi.nlm.nih.gov/gene

5 http://brassicadb.org/brad/
