# Supplementary material for: Knockout of MULTI-DRUG RESISTANT PROTEIN 5 Genes Lead to Low Phytic Acid Contents in Oilseed Rape
Source: Front Plant Sci. 2020 May 26;11:603. doi: 10.3389/fpls.2020.00603 (PMC7264376; doi:10.3389/fpls.2020.00603)

Knockout of *MULTI-DRUG RESISTANT PROTEIN 5* genes lead to low phytic acid contents in oilseed rape

Niharika Sashidhar[1], Hans J Harloff[1] and Christian Jung[1, 2]

Supplementary information

Supplementary Figure 1: Gene structure of *BnMRP5* paralogs. (a) Exon intron structure of identified *BnMRP5* genes. Exons are indicated in white boxes. C motif, Walker A and B motifs are shown in yellow, orange, and blue boxes, respectively. Black line indicates the conserved sequences between the paralogs. (b) Conserved protein sequence of BnMRP5 motifs.

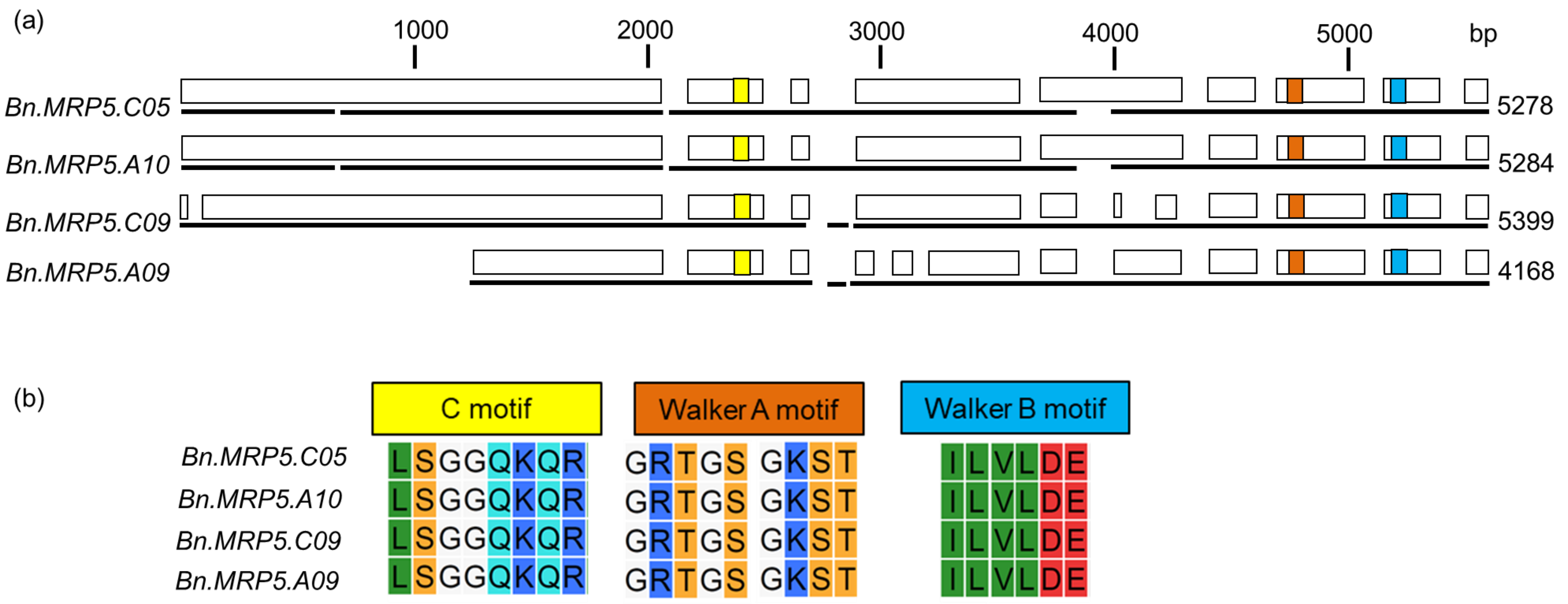

Supplementary Figure 2: Alignment of MRP5 proteins. Green boxes represent the putative transmembrane spanning domains according to Gaedeke et al. 2001. C motif, Walker A and B motifs are shown in yellow, orange, and blue boxes, respectively. Black line indicates the conserved sequences between the paralogs. Each transmembrane domain is indicated with a number along with their respective sequence from AtMRP5. Bn.MRP5.A09 lacks most of the transmembrane region resulting in lower protein size as compared to the other BnMRP5 proteins.

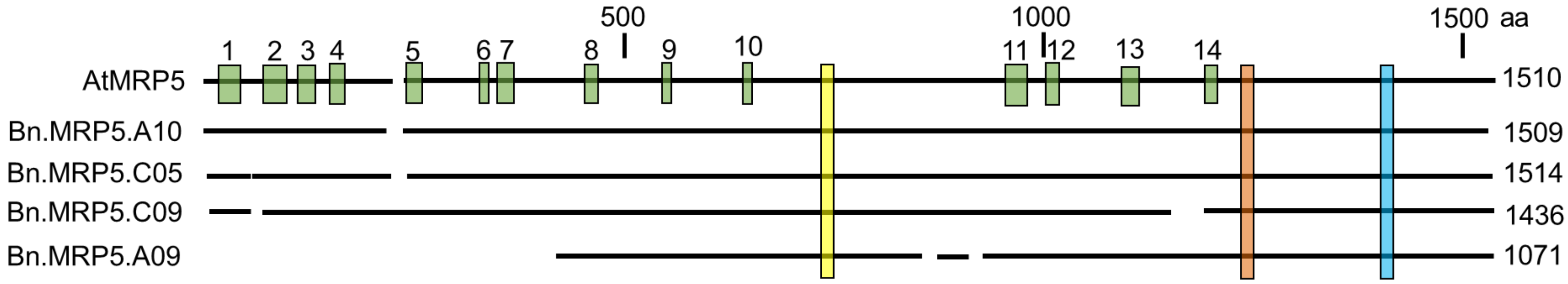

(1) LCSVIINLLLFLVFLFAVSARQILVCVRRGRDRLSKDDTVSA

(2) HVSVGFGFNLSLLCCLYVLGVQVLVLVYDGV

(3) WFVLCFPASQSLAWFVLSFLVLHL

(4) LVRIWWFLAFSICLCTMYV

(5) PYSTAGLVSLITLSWLDPLLSAG

(6) EAACNAVFAGLNTLVSYVGPYLI

(7) IFPHEGYVLAGIFFTSKLIETV

(8) IAAVATLVATIISILVTIPLA

(9) FIFWSSPIFVAAVTFATSIFLGT

(10) PTLSGIQMKVEKGMRVAVCGTVG

(11) GALIPLIILAQAAFQFLQIAS

(12) LLLIVYTALAFGSSVFIFV

(13) VMTNVTWQVFLLVVPVAVAC

(14) LLSTLVFAFCMVLLVSFP

Supplementary Figure 3: Phytic acid pathway in plants (modified after Raboy et al (2009); Desai et al (2014); Sashidhar et al (2020)). The grey color box indicates the Loewus pathway, which is a housekeeping pathway for various *myo*-inositol dependent pathways. Blue and red arrows indicate the lipid independent pathway and lipid dependent pathway, respectively. The mutagenized gene (MRP5) is highlighted in green. MIPS: *myo*-inositol phosphate synthase, MIK: *myo*-inositol kinase, IMP: *myo*-inositol mono phosphatase, 2-PGK2: 2-phosphoglyceric acid kinase, ITPK: inositol tetrakis-phosphate kinase, IPK2: inositol multi phosphate kinase, IPK: inositol pentakis-phosphate 2-kinase, MRP5: multi drug resistance protein, DAG: Diacyl glycerol, PLC: phospholipase C, PtdIS: phosphatidyl inositol synthase, Ins P_7 and Ins P_8 are pyrophosphates, Vip1 and Vip2 are inositol hexaphosphate kinases.

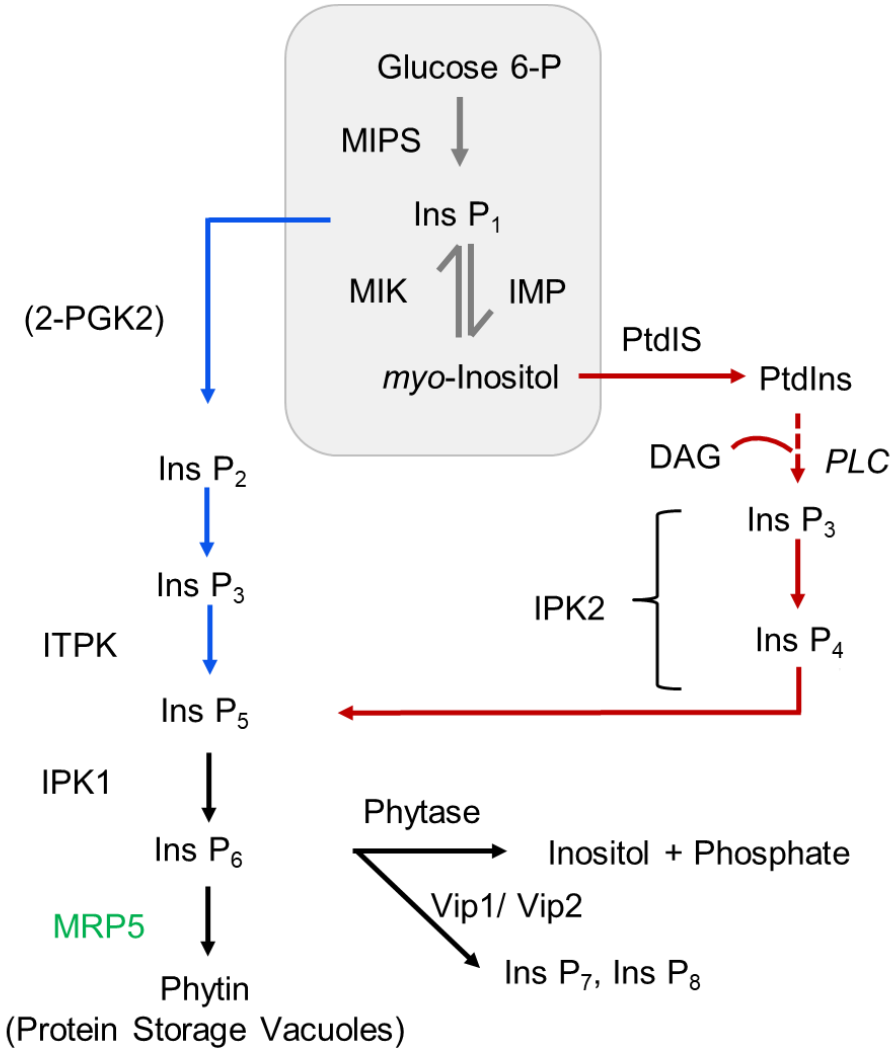

Supplement: Supplementary file 1 [file Data_Sheet_1.pdf]
